# Transgenerational Obesity and Alteration of ARHGEF11 in the Rat Liver Induced by Intrauterine Hyperglycemia

**DOI:** 10.1155/2019/6320839

**Published:** 2019-09-12

**Authors:** Wanyi Zhang, Rina Su, Hui Feng, Li Lin, Chen Wang, Huixia Yang

**Affiliations:** ^1^Department of Obstetrics and Gynecology, Peking University First Hospital, Beijing, China; ^2^Beijing Key Laboratory of Maternal Fetal Medicine of Gestational Diabetes Mellitus, Beijing, China

## Abstract

It is understood that intrauterine hyperglycemia increases the risk of obesity and diabetes in offspring of consecutive generations but its mechanisms remain obscure. This study is aimed at establishing an intrauterine hyperglycemia rat model to investigate the growth and glycolipid metabolic characteristics in transgenerational offspring and discuss the effects of Rho guanine nucleotide exchange factor 11 (ARHGEF11) and the PI3K/AKT signaling pathway in offspring development. The severe intrauterine hyperglycemia rat model was caused by STZ injection before mating, while offspring development and glycolipid metabolism were observed for the following two generations. The expression of ARHGEF11, ROCK1, PI3K, and AKT was tested in the liver and muscle tissue of F2 offspring. The results showed severe growth restriction in F1 offspring and obesity, fatty liver, and insulin resistance in female F2 offspring, especially the offspring of female intrauterine hyperglycemia-exposed parents (F2G♀C♂) and both (F2G♀G♂). The expression of ARHGEF11 and ROCK1 was significantly elevated; PI3K and phosphorylation of AKT were significantly decreased in liver tissues of F2G♀C♂ and F2G♀G♂. Our study revealed that intrauterine hyperglycemia could cause obesity and abnormal glycolipid metabolism in female transgenerational offspring; the programming effect of the intrauterine environment could cause a more obvious phenotype in the maternal line. Further exploration suggested that increased expression of ARHGEF11 and ROCK1 and the decreased expression of PI3K and phosphorylation of AKT in the liver could be responsible for the abnormal development in F2 offspring.

## 1. Introduction

Growing evidence has proved that the incidence of multiple diseases in adulthood is closely related to nutritional conditions and environmental exposure early in life, which developed into a new branch of scientific knowledge known as the developmental origins of health and disease (DOHaD) [1]. Gestational diabetes mellitus (GDM), one of the most common serious medical complications of pregnancy, has been confirmed to place offspring at an increased risk for long-term adverse outcomes including obesity and type 2 diabetes mellitus [2–4]. However, the mechanisms of intrauterine hyperglycemia affecting the glucolipid metabolism of offspring are still under discussion [5, 6]; this study is aimed at providing a basis for future research to explore the impact of intrauterine hyperglycemia on two generations of offspring and its corresponding mechanisms.

Rho guanine nucleotide exchange factor 11 (ARHGEF11) is an activator of Rho GTPases that plays a fundamental role in the regulation of G protein signaling and a number of cellular processes, including insulin secretion, insulin signaling, and lipid metabolism. Numerous studies have confirmed the correlation between a R1467H variant in ARHGEF11 and type 2 diabetes [7–11]. Rho protein kinase (ROCK), a serine/threonine (Ser/Thr) kinase, is the predominant and most direct effector molecule downstream of Rho GTPases [12, 13], and it can directly affect the Ser/Thr phosphorylation of the insulin receptor substrate (IRS) and regulate insulin resistance through the PI3K/AKT signaling pathway [14, 15]. Several studies have confirmed that ARHGEF11 affects the metabolism of glucose and fatty acids through the insulin signaling pathway and acts as a key determinant of metabolism- and obesity-associated pathologies [16–18]. In our previous work, we demonstrated the connection of ARHGEF11 and the insulin signaling pathway in the placenta with fetal macrosomia [19], with the intention of taking further our understanding of its role on the development of intrauterine hyperglycemia offspring.

In this study, we established a severe intrauterine hyperglycemia rat model and tested the glycolipid metabolism of two generations of offspring and investigated the expression of ARHGEF11, PI3K, and AKT in the dominant metabolic organs: the liver and muscle. We anticipate to provide additional evidence in the exploration of intrauterine hyperglycemia affecting offspring development and metabolism mechanisms.

## 2. Materials and Methods

### 2.1. Animal and Tissue Isolation

**Figure 1:**
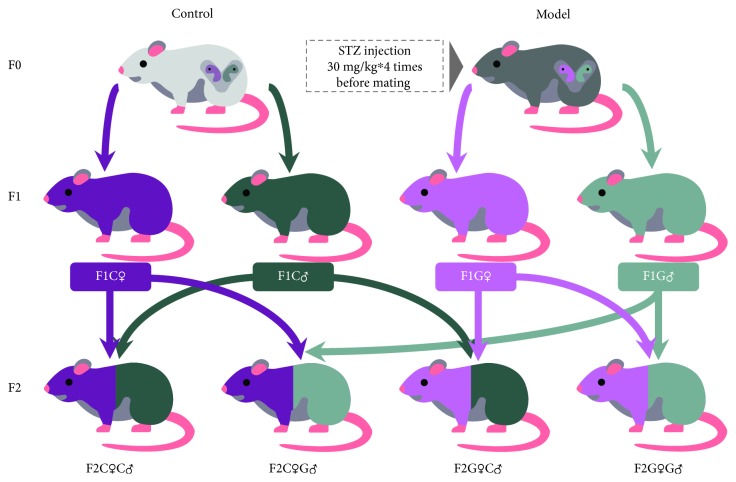
The intrauterine hyperglycemia rat model.

Wistar rats (Vital River Laboratory Animal Technology Co., Ltd., Beijing, China) were used for this study. Rats were housed in specific pathogen-free (SPF) animal rooms under a 12-hour light/dark cycle. All animal protocols were reviewed and approved by the Institutional Animal Care and Use Committee of Peking University First Hospital (J201406). At 10 weeks old, the female rats were randomly divided into two groups: the control group (F0C, *n* = 10) and the gestational diabetes mellitus group (F0G, *n* = 10). After a 12 h fast, rats in the diabetic group received an intraperitoneal injection of 2% streptozotocin (30 mg/kg, STZ, Sigma-Aldrich). The STZ injection procedure was repeated 4 times every 24 h. The control rats received an equal volume of citrate buffer. Hyperglycemia was confirmed by measuring blood glucose concentration via the tail vein. After the blood glucose of female rats reached 20-30 mmol/L, which constitutes severe hyperglycemia, the female rats mated with the normal male rats. The onset of pregnancy was determined by the presence of a copulation plug after overnight mating (designated as day 0 (D0) of pregnancy). Eight female control group and seven diabetic group rats were confirmed pregnant. The pregnant rats were allowed to deliver spontaneously. The offspring (F1) were fed a normal diet after weaning and were randomly divided into four groups: control group female offspring (F1C♀, *n* = 10), control group male offspring (F1C♂, *n* = 10), diabetic group female offspring (F1G♀, *n* = 10), and diabetic group male offspring (F1G♂, *n* = 10). The 10-week-old F1 offspring were then mated with each other and delivered four groups of F2 offspring: F2C♀C♂, F2C♀G♂, F2G♀C♂, and F2G♀G♂. The F2 offspring were fed a normal diet after weaning, and 8-10 offspring were randomly selected from each group (Figure 1). The phenotype and fasting glucose levels of F2 offspring were then characterized, and the rats were harvested at 20 weeks. Blood samples were collected from the aorta ventralis and then were centrifuged at 3000 rpm for 10 min to separate the serum. All the samples were stored at -80°C until analysis. The total triglyceride (TG), total cholesterol (TCHO), high-density lipoprotein (HDL), and low-density lipoprotein (LDL) were tested in F2 offspring in a fully automatic biochemical analyzer. Heart, liver, pancreas, kidney, and fat pads, including mesenteric fat (hereinafter “MF”), perirenal fat (hereinafter “RF”), and peripheral ovarian fat (hereinafter “OF”), were carefully dissected and weighed. The tissues were immediately frozen in liquid nitrogen and stored at -80°C.

### 2.2. HE Staining

Liver and muscle tissues were immersed in a 4% paraformaldehyde solution for 4 h and transferred to 70% ethanol. Individual lobes of tissue biopsy material were placed in processing cassettes, dehydrated through a serial alcohol gradient, and embedded in paraffin wax blocks. Prior to staining, 5 *μ*m thick tissue sections were dewaxed in xylene, rehydrated through decreasing concentrations of ethanol, washed in PBS, and then stained with hematoxylin and eosin (H&E). Post staining, sections were dehydrated through increasing concentrations of ethanol and xylene.

### 2.3. RNA Isolation and Real-Time PCR

Total RNA was isolated from liver and muscle specimens using TRIzol reagent (Invitrogen, Carlsbad, CA, USA). cDNA was synthesized using a High-Capacity cDNA Reverse Transcription Kit (Applied Biosystems, Foster, CA, USA) according to the manufacturer's recommendations. The cDNA expression levels were quantified in real-time PCR with SYBR Green Select PCR Master Mix (Applied Biosystems, Foster, CA, USA) according to the manufacturer's protocol. Real-time PCR was performed with the ABI PRISM 7500 Sequence Detector System (Applied Biosystems, Foster, CA, USA). All reactions were performed in triplicate. Statistical analysis of the results was performed with the ΔCt value (Ct gene of interest—Ct*β-actin*). The ΔCt method of relative quantification was used to determine the fold change in the expression [20]. Primers were designed using Primer Express 5.0 software. The following primers were used—*Arhgef11*: forward 5′ GCC AGC CCT CTG ACA CTT CT 3′, reverse 5′ CCA TGC TGG TCC TTT TGG AT 3′; *Rock1*: forward 5′ CAG TTG GTT CTG CCT GCA TTC 3′, reverse 5′ GCT GCT CAC CAC AAC ATA CTG 3′; *Pi3k*: forward 5′ CGA GAG TAC GCT GTA GGC TG 3′, reverse 5′ AGA AAC TGG CCA ATC CTC CG 3′; *Akt*: forward 5′ GCT TCT TTG CCA ACA TCG TG3′, reverse 5′ CAC ACA CTC CAT GCT GTC ATC T 3′; and *β-actin*: forward 5′ AGC CAT GTA CGT AGC CAT CC 3′, reverse 5′ GCT GTG GTG GTG AAG CTG TA 3′.

### 2.4. Western Blotting

Liver and muscle tissues were extracted, and Western blotting was performed as previously described [21]. Soluble cytoplasmic proteins were extracted by incubating the homogenized placental tissues with lysis buffer (RIPA with 1 mM phenylmethanesulfonyl fluoride (PMSF) (Sigma-Aldrich, St. Louis, MO, USA), 1 mM NaF, and 1 mM Na_3_VO_4_ (Shanghai Macklin Biochemical Co., Shanghai, China), and the concentration was measured with a bicinchoninic acid protein assay. Then, 40 *μ*g of total protein extract was subjected to 8% sodium dodecyl sulfate polyacrylamide gel electrophoresis (SDS-PAGE) and transferred to a nitrocellulose membrane (Amersham Pharmacia Biotech, Buckinghamshire, UK). After blocking with 5% skim milk or BSA in tris-buffered saline Tween 20 (TBST) for 1 h at room temperature, the membranes were probed with primary antibodies directed against human ARHGEF11 (dilution, 1 : 500; cat. no. ab110059; Abcam, Cambridge, UK), ROCK1, PI3K (dilution, 1 : 1000; cat. no. 4035, 4257; Cell Signaling Technology, Boston, MA, USA), AKT, and phosphor AKT (dilution, 1 : 500; cat. no. ab8805, ab38449; Abcam, Cambridge, UK) overnight at 4°C. The membranes were then incubated with horseradish peroxidase streptavidin-linked secondary antibodies (dilution, 1 : 5000; cat. no. ZB-2301; Zhongshan Golden Bridge Biotechnology Co., Beijing, China) for 1 h at room temperature and exposed to enhanced chemiluminescence reagent (ECL from Millipore, Billerica, MA, USA). Western blotting data were standardized against *β*-actin, and the relative density was analyzed with Quantity One software (Bio-Rad Laboratories Pty., Australia) and presented as the mean ± standard deviation.

### 2.5. Statistics Analyses

All values are presented as the mean ± SEM. Equality of variances and the normal distribution of errors were examined before the analysis. Statistical comparisons between groups were performed using the one-way ANOVA Kruskal-Wallis test with LSD multiple comparisons. The results were statistically analyzed by SPSS 20.0, and the statistical significance was defined as *p* < 0.05.

## 3. Results

### 3.1. Lower Body Weight and Abnormal Glucose Tolerance in F1 Offspring

After the injection of STZ in F0 pregnant rats causing severe hyperglycemia during pregnancy, the resulting body weight of the F1G group demonstrated in a significant decrease in both male and female offspring ([Supplementary-material supplementary-material-1]). Compared to F1C, F1G rats possessed significantly abnormal glucose tolerance, while the area under the curve (AUC) of OGTT was significantly lower in F1G than F1C rats when measured at 4, 12, 16, and 20 weeks and the serum insulin was significantly higher in F1G rats when measured at 8, 12, and 16 weeks ([Supplementary-material supplementary-material-1]).

### 3.2. Higher Body Weight and Fat Accumulation in Female F2 Offspring

**Figure 2:**
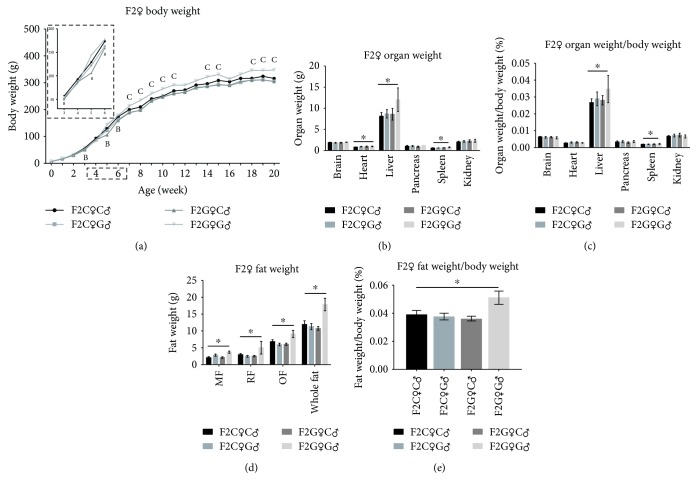
The body weight and organ weight of female F2 offspring rats. MF: mesenteric fat; RF: perirenal fat; OF: peripheral ovarian fat. (a) F2C♀C♂ vs. F2C♀G♂, *p* < 0.05, (b) F2C♀C♂ vs. F2G♀C♂, *p* < 0.05, and (c) F2C♀C♂ vs. F2G♀G♂, *p* < 0.05 and ^∗^*p* < 0.05.

In the male F2 offspring, we did not see meaningful changes in body weight and organ weight ([Supplementary-material supplementary-material-1]). Although there are some differences in glucose tolerance and lipid levels between the male F2 offspring rat at different ages, these differences are not concentrated in a certain group ([Supplementary-material supplementary-material-1]). Meanwhile, the body weight, organ weight, and fat weight varied significantly between different female F2 offspring groups. The F2G♀C♂ group recorded a respective higher body weight when compared to the three other groups at 3, 5, and 6 weeks old, while the F2G♀G♂ group consistently recorded higher body weight since week 7 (Figure 2(a)). The relative weight of the livers and spleens in the F2G♀G♂ group was significantly higher than those in the F2C♀C♂ group (Figures 2(b) and 2(c)). The weights of MF, RF, OF, and whole fat of the F2G♀G♂ group rats were significantly higher than those of the control group rats (Figures 2(d) and 2(e)).

### 3.3. Abnormal Metabolism of Glucose and Lipid in Female F2 Offspring

**Figure 3:**
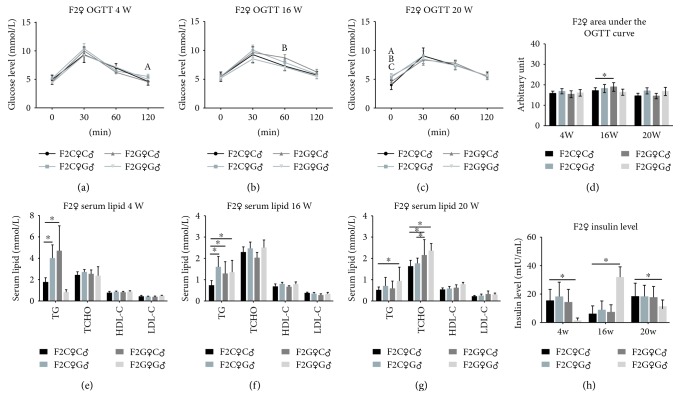
The OGTT, serum lipid, and insulin of female F2 offspring rats. (a) F2C♀C♂ vs. F2C♀G♂, *p* < 0.05, (b) F2C♀C♂ vs. F2G♀C♂, *p* < 0.05, and (c) F2C♀C♂ vs. F2G♀G♂, *p* < 0.05 and ^∗^*p* < 0.05.

For testing the glucose tolerance of the F2 offspring, the OGTT test was performed at 4, 16, and 20 weeks. Our results, as listed in Figures 3(a)–3(c), showed that at 4 weeks, the 2 h plasma glucose for the F2C♀G♂ group was significantly higher than that for the F2C♀C♂ group. At 16 weeks, the 1 h plasma glucose of the F2G♀C♂ group was significantly higher than that of the F2C♀C♂ group. At 20 weeks, the FPG in the F2C♀G♂, F2G♀C♂, and F2G♀G♂ groups was significantly higher than that in the F2C♀C♂ group. The AUC of OGTT in the F2G♀C♂ group at 16 weeks was significantly larger than that in the F2C♀C♂ group (Figure 3(d)). The serum lipid levels at 4, 16, and 20 weeks were also tested. The serum TG in three intrauterine hyperglycemia F2 groups showed varying degrees of increase when measured at 4, 16, and 20 weeks (Figures 3(e)–3(g)). The TCHO in the F2G♀C♂ and F2G♀G♂ groups was significantly higher than that in the F2C♀C♂ group at 20 weeks (Figure 3(g)). The insulin levels failed to show a consistent trend. The serum insulin in the F2G♀G♂ group was significantly lower at 4 and 20 weeks, but singularly higher at 16 weeks (Figure 3(h)).

### 3.4. Hepatic Pathological Changes in Female F2 Offspring

**Figure 4:**
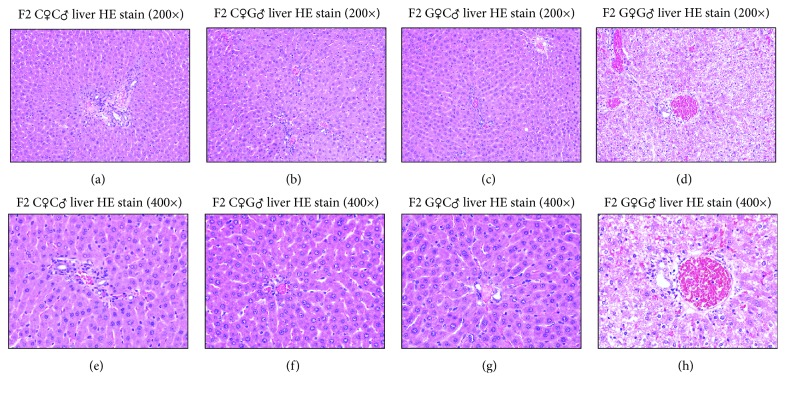
The liver HE stain of female F2 offspring rats.

After observing liver hypertrophy in the F2G♀G♂ group, the tissue was stained with HE for further research. Under the microscope, the nucleus of the liver cells in the F2G♀G♂ group appeared to be surrounded by different sizes of vacuoles (lipid droplet) (Figure 4(h)), while the F2C♀G♂ group and F2G♀C♂ group did not show a significant difference when compared with the F2C♀C♂ group.

### 3.5. ARHGEF11 and Insulin Signaling in the Liver and Muscle of Female F2 Offspring

**Figure 5:**
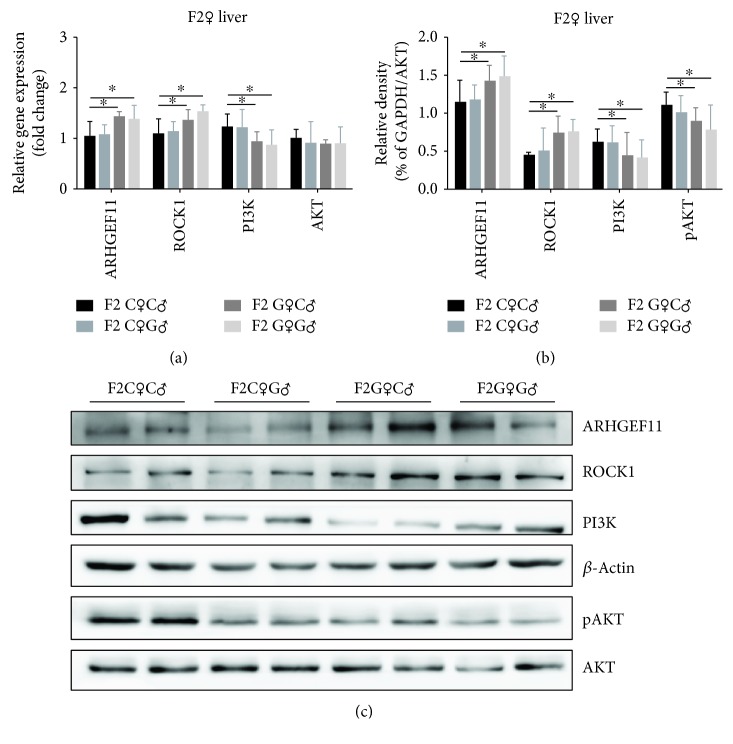
The gene and protein expression of metabolism signaling in the liver of female F2 offspring rats. ^∗^*p* < 0.05.

**Figure 6:**
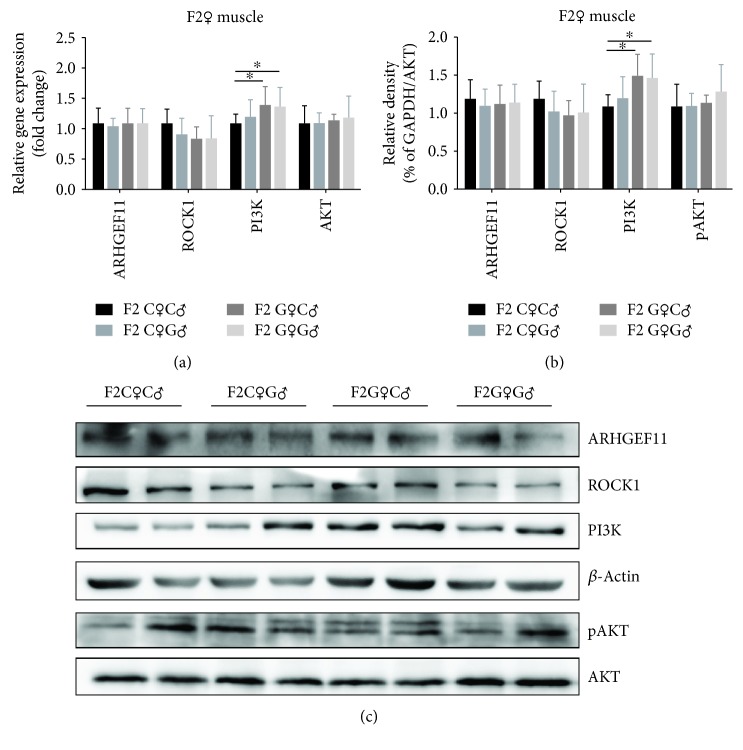
The gene and protein expression of metabolism signaling in the muscle of female F2 offspring rats. ^∗^*p* < 0.05.

In order to further our understanding about the dependent variables of obesity in F2 offspring, we tested the gene and protein expression of metabolism signaling in the liver and muscles. In the liver, the expression of ARHGEF11 and ROCK1 was significantly increased in the F2G♀C♂ and F2G♀G♂ groups and PI3K was significantly decreased in these two groups in both gene and protein expressions (Figures 5(a)–5(c)). The gene expression of *Akt* showed no significant difference between the four groups, but the phosphorylation of AKT (pAKT) was significantly suppressed in the F2G♀C♂ and F2G♀G♂ groups (Figures 5(a)–5(c)). In the muscles, the gene and protein expressions of ARHGEF11, ROCK1, and AKT showed no significant difference between the four groups but PI3K was significantly increased in the F2G♀C♂ and F2G♀G♂ groups (Figures 6(a)–6(c)). The gene expression of *Arhgef11*, *Rock1*, *Pi3k*, and *Akt* in the liver and muscles of F1 offspring did not show a significant change ([Supplementary-material supplementary-material-1]).

## 4. Discussion

Here, we established that severe hyperglycemia during pregnancy could lead to obesity and increased risk of metabolism syndrome in female F2 offspring rats by affecting the expression of ARHGEF11 and insulin signaling molecules in the liver and muscles. The F2 offspring of maternal intrauterine hyperglycemia-exposed rats demonstrated significantly higher body weight at 3, 5, and 6 weeks when compared to the control group, while F2 offspring of both intrauterine hyperglycemia-exposed parents demonstrated significant obesity since week 7. In addition, the serum glycolipid characteristics in female F2 offspring showed an abnormal change; the microscopy of the liver showed significant hepatic steatosis. The expression of ARHGEF11 was significantly higher in the livers of the F2G♀C♂ and F2G♀G♂ groups, and insulin signaling molecules were accordingly suppressed, which may explain an influential factor for obesity in F2 offspring. However, the expression of ARHGEF11 and insulin signaling molecules in muscle tissues showed an opposing trend, which suggests complex regulation patterns of obesity and metabolism in the offspring of gestational hyperglycemia parents.

It was anticipated that the F2 offspring of intrauterine hyperglycemia-exposed F1 offspring recorded higher body weight and abnormal metabolism of glycolipids. On one hand, there were clinical reports about the risk of obesity and diabetes in adult offspring of malnourished parents [22, 23] and they constantly presented the most severe phenotype in F2 offspring whose parents were both pregnant under abnormal intrauterine nutrition circumstances. Besides that, there are existing, published animal models that document nutritional state changes during pregnancy that increase the risk of obesity and abnormal glucose metabolism in F2 offspring [24, 25], which were consistent with our results. It is worth noting that, in this study, the significance in obesity only appeared in female F2 rats, which may be related to estrogen-induced body fat accumulation. However, there are other researches that showed that high-fat diet-induced obese male parents led to a 67% increase in fat in F1 females and a 24% increase in fat in F2 males, which had no consistent gender bias [26]. These conclusions may provide insights for differential expression studies of the role of paternal programming in sex chromosomes.

Furthermore, this study revealed the differences in the effects of abnormal metabolism from female and male parents on offspring. Whether in the weight, glucose tolerance index, and expression of insulin signaling pathways in peripheral metabolic tissues, aside the most obvious F2G♀G♂ group, F2G♀C♂ were more severely affected than F2C♀G♂. This result may provide some theoretical basis for the programming effect of the intrauterine environment on the growth and development of the offspring. However, previous studies suggest that the adverse intrauterine environment has a more significant influence than the paternal line [26, 27]. The analysis of two generations of famine-exposed populations in the Netherlands found that the body weight of the F2 offspring whose father was famine-exposed was significantly higher than that of the control group, while no significant difference was found in the F2 offspring whose mother was famine-exposed [22, 23]. Martínez et al. found that intrauterine adverse effects on the environment by affecting the methylation of the sperm LXRA gene in F1 male hamsters led to glucose intolerance in F2 hamsters [28]. Differences in these results may be due to differences in model construction and differences in F1 developmental phenotypes. Further understanding of maternal and parental factors affecting the offspring requires additional large-sample studies to investigate.

In the exploration of impact factors of F2 offspring development, we measured the expression of ARHGEF11, ROCK1, PI3K, and AKT in the liver and muscle tissues both in F1 and in F2 offspring. Rho GTPases are highly conserved signaling proteins that regulate many essential cellular processes, including cytoskeletal dynamics, cell cycle progression, adhesion, migration, and vesicle trafficking [29, 30]. Sophisticated processing is regulated by more than 80 GEFs and 70 GAPs [31, 32]. ARHGEF11 functions to activate Rho by interacting with Ga12 and Ga13 [33]. The relationship between ARHGEF11-activated Rho with leukemia, prostate cancer, and embryonic development and modulating insulin signaling was sequentially reported [17, 34–36]. Regulation of ARHGEF11 on IRS1 signaling and the correlation with type 2 diabetes are important for glucose and fatty acid metabolism. Insulin signaling in target tissues is essential for growth and development as well as for normal homeostasis of glucose, fat, and protein metabolism. Ser/Thr phosphorylation of IRS1 is a key negative feedback that uncouples IRS1 proteins from their upstream and downstream effectors and terminates signal transduction in response to insulin [37, 38]. Tyr-phosphorylated IRS1 combined with PI3K activates 3-phosphoinositide-dependent protein kinase 1, which promotes the phosphorylation of AKT, and the phosphorylation of AKT activates several signaling molecules involving glucose metabolism [39].

The activation of ARHGEF11 and ROCK1, as well the decreased expression of PI3K and phosphorylation of AKT in the liver tissue of the F2G♀C♂ and F2G♀G♂ groups, indicated a decrease in hepatic glucose metabolism. The expression of metabolism molecules was consistent with the OGTT results in these two groups, suggesting that this might be the cause of obesity and insulin resistance in F2 offspring. The results in the muscle tissue failed to show a consistent trend, and the higher expression of PI3K may indicate an increase in compensatory glucose metabolism in muscles.

However, after STZ injections, the F0 rat developed severe hyperglycemia during pregnancy, which led to lower body weight and abnormal lower blood glucose levels in F1 offspring. Considering the very short half-life of STZ (approximately 15 min), it is unlikely that its direct effects could be responsible for the growth restriction of F1 offspring [40]. The intrauterine hyperglycemia model caused by STZ injections before pregnancy was similar with the type 1 and type 2 diabetes pregnant patients, which often results in offspring overgrowth or growth hindrances under different degrees of maternal hyperglycemia [41]. Although other researches have demonstrated that growth restriction of diabetes pregnant patients' offspring suffered a higher risk of subsequent obesity and type 2 diabetes [42, 43], both female and male F1 offspring in this study remained having a lighter body weight until week 20, with a smaller area under the OGTT curve and higher insulin levels at 12 and 16 weeks ([Supplementary-material supplementary-material-1]). And the gene expression of *Arhgef11*, *Rock1*, *Pi3k*, and *Akt* in the liver and muscle of F1 offspring did not show a significant change ([Supplementary-material supplementary-material-1]). This result was speculated due to severe intrauterine hyperglycemia leading to severe developmental limitation in F1 offspring. Follow-up research with high-calorie diets may have better findings regarding the metabolic injuries of F1 offspring.

In conclusion, this study established a severe intrauterine hyperglycemia rat model and explored its impact on two generations of offspring. The results showed that severe intrauterine hyperglycemia could lead to growth restriction in F1 offspring and cause obesity, fatty liver, and insulin resistance in female F2 offspring, particularly the offspring with female growth restriction parents (F2G♀C♂) and both (F2G♀G♂). Further research suggested that the increased expression of ARHGEF11 and ROCK1 and the suppressive expression of PI3K and phosphorylation of AKT in the liver could be responsible for the abnormal development in F2 offspring. These results indicated that intrauterine hyperglycemia could cause severe metabolism and development problems in two generations of offspring and was closely related to hepatic insulin signaling pathways.

## Data Availability

The data used to support the findings of this study are available from the corresponding author upon request.

## Supplementary Materials

Supplementary Materials Fig S1: the body weight and glucose tolerance of F1 offspring rats. ^∗^*p* < 0.05. Fig S2: the body weight and glucose tolerance of male F2 offspring rats. Fig S3: the OGTT, serum lipid, and insulin of male F2 offspring rats. Fig S4: the gene expression of metabolism signaling in the liver and muscle of F1 offspring rats.
