# Supplementary material for: Transgenerational Obesity and Alteration of ARHGEF11 in the Rat Liver Induced by Intrauterine Hyperglycemia
Source: J Diabetes Res. 2019 Sep 12;2019:6320839. doi: 10.1155/2019/6320839 (PMC6757444; doi:10.1155/2019/6320839)
Supplement: Supplementary Materials — Fig S1: the body weight and glucose tolerance of F1 offspring rats. ∗p < 0.05. Fig S2: the body weight and glucose tolerance of male F2 offspring rats. Fig S3: the OGTT, serum lipid, and insulin of male F2 offspring rats. Fig S4: the gene expression of metabolism signaling in the liver and muscle of F1 offspring rats. [file 6320839.f1.doc]

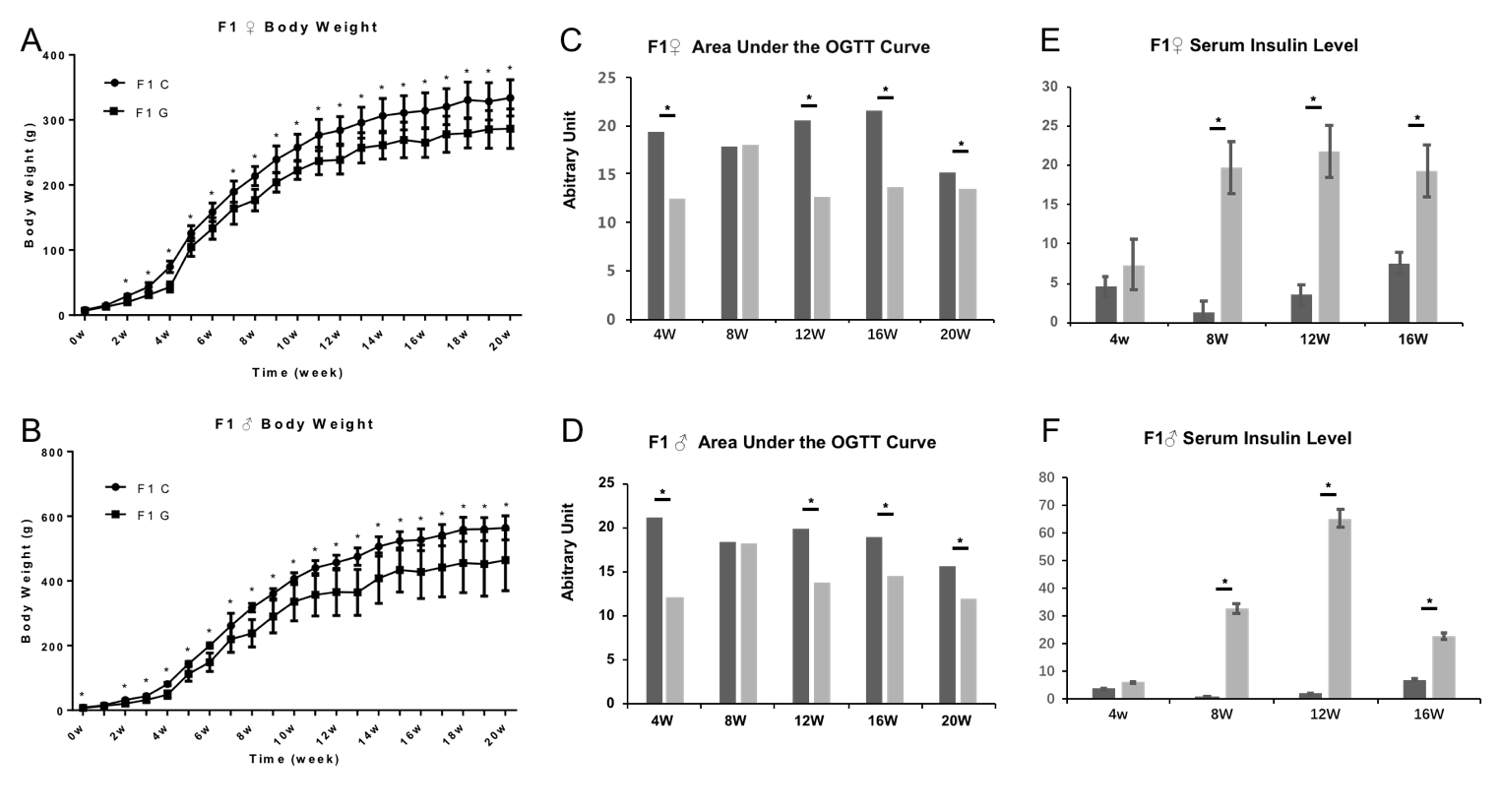

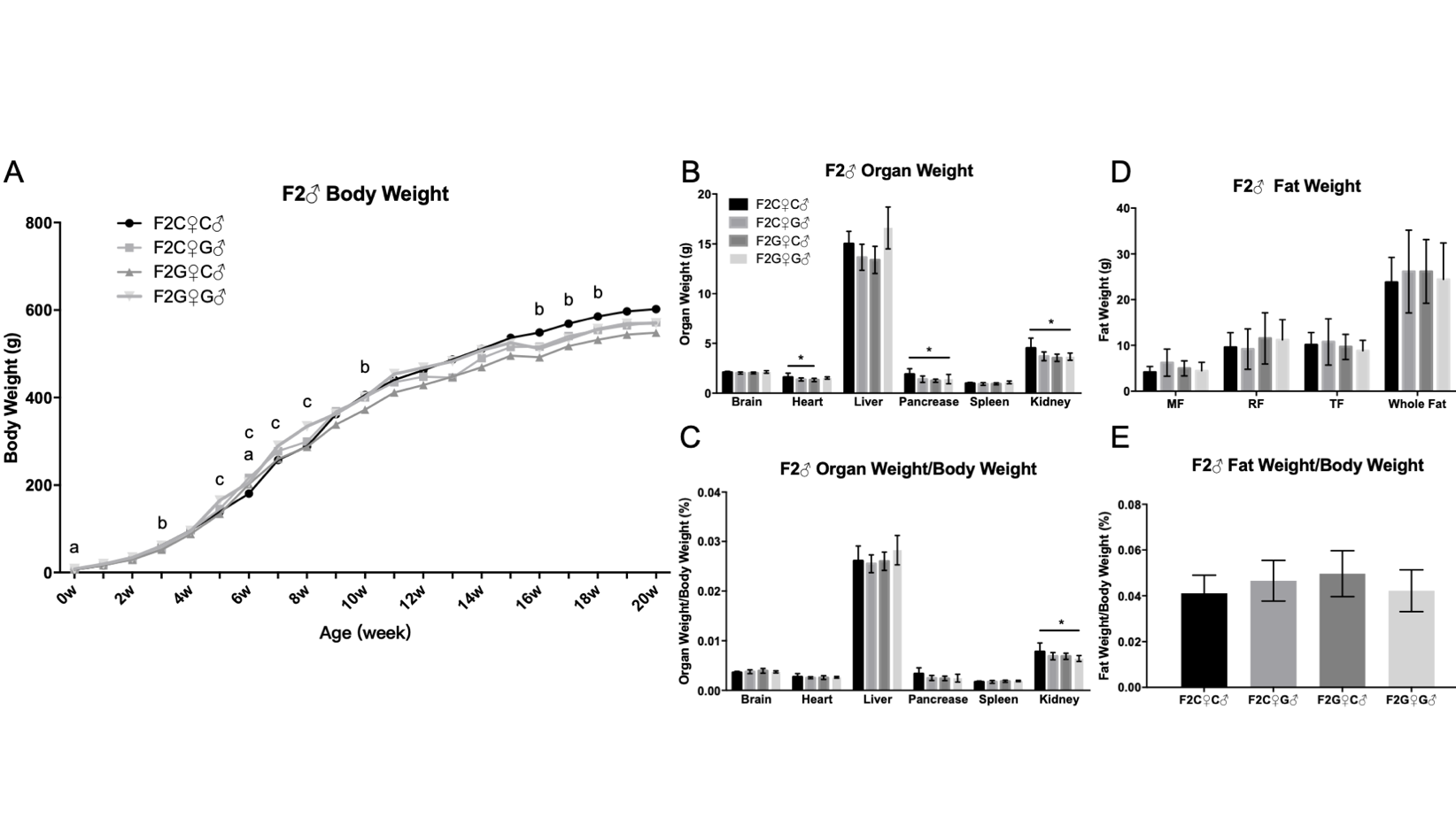
 Fig S1. The body weight and glucose tolerance of F1 offspring rat. *p＜0.05.

Fig S2. The body weight and glucose tolerance of male F2 offspring rats.

MF: mesenteric fat, RF: perirenal fat, TF: peripheral testicular fat.

a: F2C♀C♂ vs F2C♀G♂ p＜0.05, b: F2C♀C♂ vs F2G♀C♂ p＜0.05, c: F2C♀C♂ vs F2G♀G♂ p＜0.05, *p＜0.05.


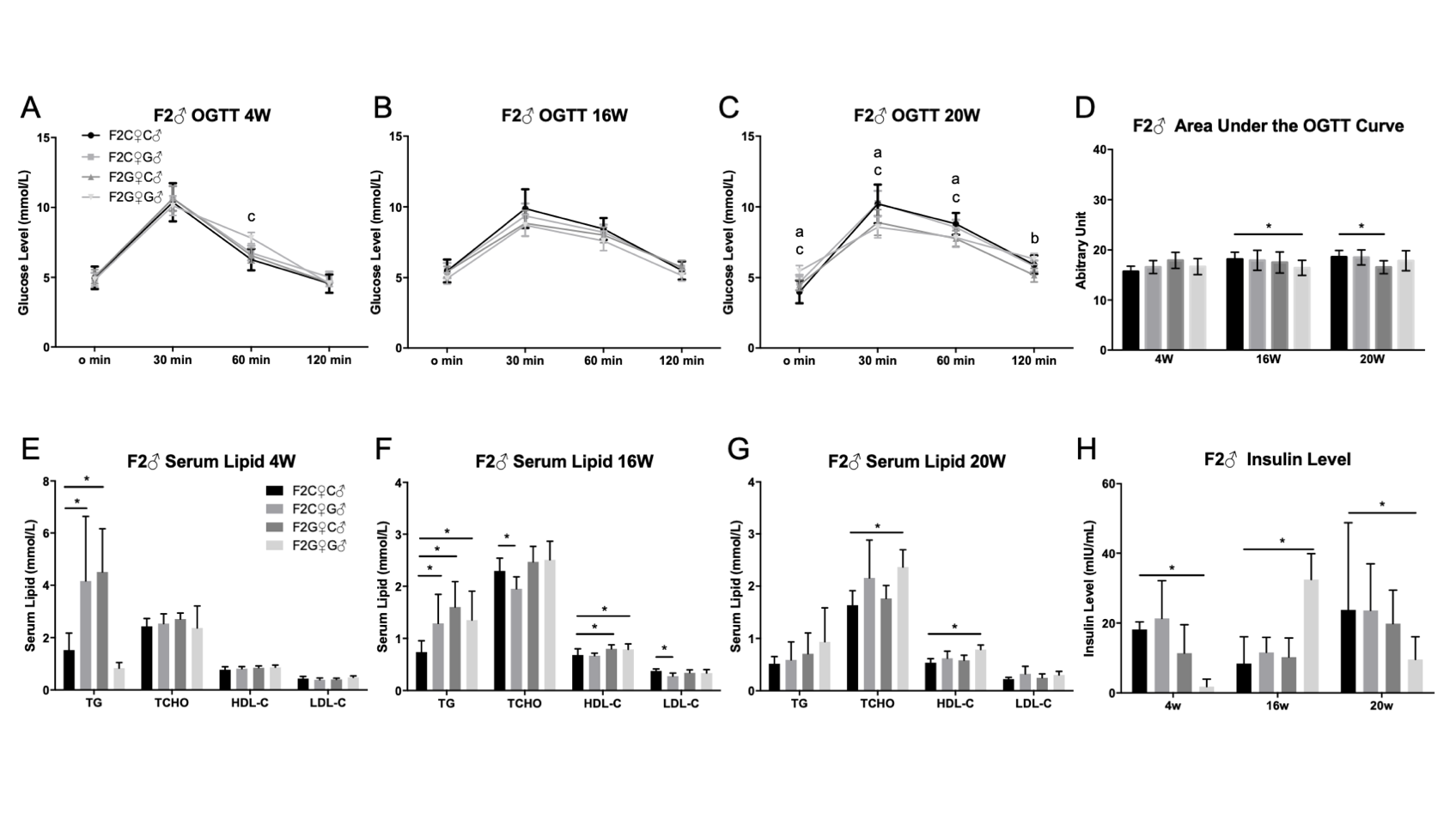
Fig S3. The OGTT, serum lipid and insulin of male F2 offspring rats.


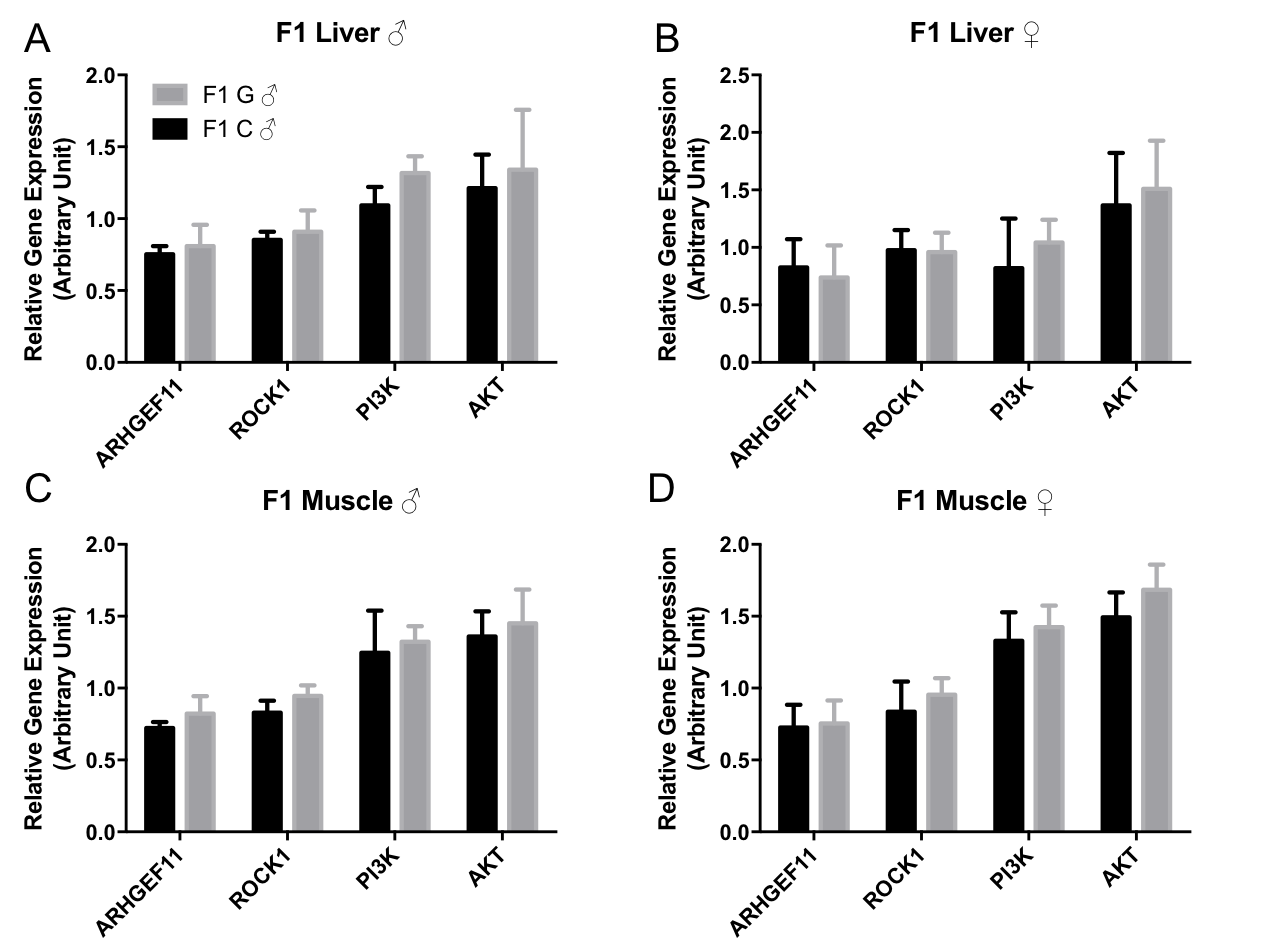
a: F2C♀C♂ vs F2C♀G♂ p＜0.05, b: F2C♀C♂ vs F2G♀C♂ p＜0.05, c: F2C♀C♂ vs F2G♀G♂ p＜0.05, *p＜0.05.

Fig S4. The gene expression of metabolism signaling in liver and muscle of F1 offspring rats.
